# Swimming energetics of Atlantic salmon in relation to extended fasting at different temperatures

**DOI:** 10.1093/conphys/coac037

**Published:** 2022-06-17

**Authors:** Malthe Hvas

**Affiliations:** Institute of Marine Research, Matre 5, 5984 Matredal, Norway

**Keywords:** tail beat frequency, sustained aerobic swimming, starvation, migration, critical swimming speed, cost of transport

## Abstract

Predicted future warming of aquatic environments could make fish vulnerable to naturally occurring fasting periods during migration between feeding and spawning sites, as these endeavours become energetically more expensive. In this study, Atlantic salmon (*Salmo salar*) acclimated to midrange (9°C) or elevated suboptimal (18°C) temperatures were subjected to critical (U_crit_) and sustained (4 hours at 80% U_crit_) swimming trials before and after 4 weeks of fasting. Fasting caused weight losses of 7.3% and 8.3% at 9°C and 18°C, respectively. The U_crit_ was unaffected by fasting, but higher at 18°C. Fatigue was associated with higher plasma cortisol, osmolality, Na^+^ and Cl^−^ at 18°C, and ionic disturbances were higher in fasted fish. All fish completed the sustained swim trials while maintaining constant oxygen uptake rates (ṀO_2_), indicating strictly aerobic swimming efforts. At low swimming speeds ṀO_2_ was downregulated in fasted fish by 23.8% and 15.6% at 9°C and 18°C, respectively, likely as an adaptation to preserve resources. However, at higher speeds ṀO_2_ became similar to fed fish showing that maximum metabolic rates were maintained. The changes in ṀO_2_ lowered costs of transport and optimal swimming speeds in fasted fish at both temperatures, but these energetic alterations were smaller at 18°C while routine ṀO_2_ was 57% higher than at 9°C. As such, this study shows that Atlantic salmon maintain both glycolytic and aerobic swimming capacities after extended fasting, even at elevated suboptimal temperatures, and adaptive metabolic downregulation provides increased swimming efficiency in fasted fish. Although, improved swimming energetics were smaller when fasting at the higher temperature while metabolism becomes elevated. This could affect migration success in warming climates, especially when considering interactions with other costly activities such as coping with parasites obtained when passing aquaculture sites during seaward travel or gonad development while being voluntarily anorexic during upriver travel to spawning grounds.

## Teaser

Fish endures prolonged fasting periods during migration, while costs of swimming increase in warming environments, which restrains energy budgets. Here, critical and sustained swimming capacities along with metabolic rates and haematological parameters were assessed in Atlantic salmon before and after 4 weeks of fasting at midrange and elevated suboptimal temperatures.

## Introduction

All physiological functions have an energetic cost, and animals therefore require food to obtain energy to offset these costs if they are to forage, grow, migrate, reproduce and survive (Porter and Gates, 1969). How often animals need to eat varies between species group. For instance, fish and other ectotherms may endure weeks or months of food deprivation without suffering detrimental effects owing to lower metabolic demands than in endotherms such as birds and mammals (McCue, 2010; Wang *et al.,* 2006).

In nature, many species of fish will periodically encounter extended fasting periods because of seasonal fluctuations in food supplies in their habitat, migratory behaviours or during reproduction events (Green and Farwell, 1971; Miller *et al.,* 2009; Van Ginneken *et al.,* 2005). To cope during periods of food shortage, fish are able to downregulate metabolic rates to preserve resources (Fu *et al.,* 2005; Hvas *et al.,* 2020; Mehner and Wieser, 1994), which on the biochemical level is facilitated by beneficial changes in gene expressions, enzyme activities and mitochondrial functions in various organs (Bermejo-Nogales *et al.,* 2015; Cassidy *et al.,* 2016; Méndez and Wieser, 1993; Salin *et al.,* 2018). Furthermore, following extended periods of food deprivation, such physiological adjustments can be reversed and stunted growth can be compensated as fish have flexible and indeterminate growth trajectories (Ali *et al.,* 2003; Hvas *et al.,* 2022; Morgan and Metcalfe, 2001).

Although fish are well adapted to temporal variability in feeding opportunities, long-term starvation will eventually jeopardize survival as physiological functions become compromised. The impact of extended fasting periods will depend on species, initial body condition and various environmental factors. Water temperature is a particularly important environmental factor to consider, as increasing temperatures drastically elevates the metabolic demands in fish (Brett, 1971; Eliason and Farrell, 2016). Warming of aquatic habitats from anthropogenic climate change could therefore make species of fish more vulnerable to naturally occurring extended fasting periods as energy stores gets depleted sooner (Morgan *et al.,* 2001).

The Atlantic salmon (*Salmo salar*) is a relevant species to study with regards to the interactions between fasting and temperature on energetic costs and physiological capacities. It is an anadromous migratory species that endures prolonged fasting periods at various phases in its life cycle (Hendry and Beall, 2004; Kadri *et al.,* 1995; Thorstad *et al.,* 2012). Furthermore, it also encounters variable thermal environments ranging from 0–3°C in its northern distribution (Lacroix, 2013; Reddin, 1985) to presumably above 20°C occasionally during summer river migrations in its southern limits (Valiente *et al.,* 2011). Although, for the majority of its life during the marine phase, it is found in waters below 10°C (Jensen *et al.,* 2014; Lacroix, 2013; Reddin, 1985). Growth of Atlantic salmon post-smolts is maximized at 13°C (Handeland *et al.,* 2003; Handeland *et al.,* 2008) and is reduced above 18°C owing to lower appetite and feed conversion efficiency (Hevrøy *et al.,* 2015; Kullgren *et al.,* 2013; Wade *et al.,* 2019). Behaviorally, Atlantic salmon will actively avoid environments above 16°C (Johansson *et al.,* 2009; Lacroix, 2013) and long-term survival is not possible at chronic temperatures above 22°C (Gamperl *et al.,* 2020; Hvas *et al.,* 2017).

Populations of Atlantic salmon have long been declining as a consequence of habitat destruction and overfishing (Dadswell *et al.,* 2021; Pardo *et al.,* 2021; Parrish *et al.,* 1998), with more recent threats coming from sea cage aquaculture that spreads parasitic copepodids onto wild seaward migrating fish (Johnsen *et al.,* 2020; Krkošek *et al.,* 2007). These parasites exert a substantial increased energetic burden on infected fish (Hvas and Bui, 2022), coinciding with observed reductions in body conditions of salmon returning from sea (Susdorf *et al.,* 2018). Such energetic disadvantages will presumably be exacerbated by increasing temperatures and make completion of life cycles more difficult.

Energetics of fish are most often assessed from oxygen uptake rates (ṀO_2_) as a proxy of the aerobic metabolic rate (Clark *et al.,* 2013; Nelson, 2016). Using swim tunnel respirometers, the ṀO_2_ can be measured during swimming at defined activity levels together with maximum swimming capacities (Brett, 1964; Steffensen *et al.,* 1984). From ṀO_2_ at known swimming speeds the cost of transport (CoT) can be derived, and the optimal swimming speed (U_opt_) can be defined as the speed that minimizes the CoT (Claireaux *et al.,* 2006). For general cruising in search of food and migration specifically, fish should swim at their U_opt_ to maximize energy efficiency, which also seems to be the case when monitoring fish movements in the wild (Drenner *et al.,* 2012; Weihs, 1973).

When assessing maximum swimming capacities, protocols most commonly focus on the highest attainable speed by incrementally increasing water currents until fatigue is reached. These are known as critical swim speed (U_crit_) tests, which involves utilization of both aerobic and anaerobic metabolism (Farrell, 2007; Wilson and Egginton, 1994). Less commonly, protocols may also focus on strictly aerobic fueled metabolism in sustained swimming trials where fish are tested at a constant speed for several hours (Beamish, 1978; Cotterell and Wardle, 2004; Hvas *et al.,* 2021a). In the case of fed Atlantic salmon within midrange temperatures, the transition from sustained aerobic swimming to a mixture of aerobic and anaerobic swimming is at 80–85% of the U_crit_, while the U_opt_ for minimum CoT tends to be 60–65% of the U_crit_ (Beddow and McKinley, 1999; Hvas *et al.,* 2021a; Hvas and Oppedal, 2017). Together, the U_crit_, the U_opt_ and sustained swimming capacity provides valuable insights into the athleticism and energetics of fish that are reflective of environmental adaptations (Beamish, 1978; Claireaux *et al.,* 2006; Plaut, 2001).

The physiological responses to extended fasting periods in Atlantic salmon have so far mainly been studied at the lower and mid ranges of their thermal niche. For instance, at 12°C Atlantic salmon gradually downregulate resting metabolic rates over a 4-week fasting period as an adaptive response to save energy, while still maintaining the ability to adequately respond and recover from acute stress (Hvas *et al.,* 2020, 2021b). Additionally, weight loss and changes in body composition during up to 12 weeks of fasting have been studied in winter conditions, showing a remarkable resilience to food deprivation at low temperatures (Einen *et al.,* 1998; Lie and Huse, 1992).

Prolonged fasting may influence aerobic and anaerobic swimming properties differently. Fast white muscle fibres tend to be more affected than slow red muscle fibres in a range of fish species investigated with regards to fibre size, protein synthesis, glycogen levels and capillary supply, and effects are larger on glycolytic than on oxidative functionality regardless of fibre type (Beardall and Johnston, 1983; Johnston and Goldspink, 1973; Loughna and Goldspink, 1984; Martínez *et al.,* 2003; Scarabello *et al.,* 1991). As such, food deprivation should first reduce high-speed short-term swimming capacity (e.g. U_crit_), while sustained aerobic swimming capacities will remain preserved for longer periods. In Atlantic cod (*Gadus morhua*) fasted for 12 weeks, the U_crit_ was indeed reduced due to impairment of anaerobic work (Martínez *et al.,* 2004). Although the U_crit_ in Atlantic salmon remained unaffected after 4 weeks of fasting at 12°C (Hvas *et al.,* 2021b), similar resilience to food deprivation is presumably not possible in warmer, less optimal environments. Moreover, it is unknown to what extent Atlantic salmon are able to downregulate metabolic demands at higher temperatures during fasting periods and how sustained swimming, CoT and U_opt_ are affected. Food-deprived fish may therefore have less capacity for long-term high-intensity swimming within their normal aerobic limit when substrate depletion is imminent.

The purpose of the present study was to compare the impact of a 4-week fasting period at a normal midrange (9°C) and an elevated suboptimal (18°C) temperature with regards to swimming energetics and capacities in Atlantic salmon. Fed and fasted fish at both temperatures were subjected to a typical U_crit_ trial as well as a 4-hour sustained swimming trial at 80% of the U_crit_. It was hypothesized that food-deprived Atlantic salmon would be more impaired at the higher temperature because accelerated metabolic rates would deplete resources more quickly. Specifically, it was predicted that fasting at 18°C would reduce U_crit_ and that fish would struggle to complete the sustained swimming trial, while fasted fish at 9°C would maintain their swimming capacities as well as benefitting from an improved CoT. Finally, analyses of haematological parameters in fatigued fish were done to assess potential differences in osmotic, endocrine and metabolic disturbances, for instance owing to changes in the functionality of anaerobic muscle fibres in fasted fish.

## Materials and methods

### Fish husbandry

Atlantic salmon post-smolts produced on site at the Matre Research Station, Institute of Marine Research, Norway, were allocated in six indoor circular holding tanks (diameter, 3 m; volume, 5.3 m^3^). The holding tanks were supplied with aerated, filtered and UV-C-treated full strength seawater of 34 ppt with a continuous inflow of 120 l min^−1^, which ensured constant oxygen levels above 85% saturation and prevented accumulation of waste products. Three tanks were maintained at 9°C and the other three tanks at 18°C. The temperature was controlled by automatic mixing of ambient and heated water supplies in large header tanks prior to reaching the holding tanks. The fish were subjected to a simulated natural photoperiod and fed commercial feed in excess each day via automated feeders (pellet size, 4.5 mm; Skretting, Norway). Before starting the experimental trials, the fish had been acclimating in these conditions for approximately 1 month.

This study was made between September and December 2021 following ethical approval by The Norwegian Food Safety Authorities under permit number 254966 for use of animals in scientific research.

### Swim tunnel setup

Critical and sustained swim trials were performed using a large Brett-type swim tunnel respirometer that has been described in detail previously (Hvas *et al.,* 2017; Remen *et al.,* 2016). The key dimensions of this system were a 248-cm-long cylindrical swim section with an internal diameter of 36 cm and a total water volume of 1905 l. A camera was fixated behind the rear grid downstream of the swim section so that the fish being tested could be observed remotely. An oxygen sensor (RINKO ARO-FT, JFE Advanced, Japan) was attached next to the camera and was setup via computer software to log oxygen concentrations in 2-second intervals (MiniSoft SD200W, SAIV Environmental Sensors & Systems, Norway). In the downstream end of the swim section the top lid could be removed for access when fish had to be transferred into or from the tunnel. To minimize turbulence and achieve approximately laminar flow conditions, a resting chamber followed by a flow straightener with honeycomb-shaped cells (5 mm in diameter) and a reduction cone was situated upstream of the swim section. Water current speeds were generated with a motor-driven propeller (Flygt 4630, 11° propeller blade, Xylem Water Solutions, Norway) opposite of the swim section, and a flow metre (Höntzsch Flow Measuring Technology, Germany) was used to confirm that desired current speeds were obtained with a given motor output. A large pipe connected to the same header tanks as used for the holding tanks supplied water into the tunnel via an inlet behind the propeller section. Here, a steady open flow through the tunnel ensured stable temperatures and normoxic conditions. However, for measurements of ṀO_2_ this water supply could be closed off periodically followed by subsequent rapid flushing to re-establish oxygen levels.

### Experimental design and protocols

In the first part of the experiment, U_crit_ and sustained swim trials were performed on fed fish acclimated to either 9°C or 18°C. After completion of these trials, feeding was stopped, and the fish were fasted in their holding tanks for 4 weeks. In the second part of the experiment, after the 4-week fasting period, U_crit_ and sustained swim trials were performed again at both temperatures.

Prior to each U_crit_ trial, eight fish were netted from an appropriate holding tank in the afternoon and quickly transferred to the swim tunnel that was located in the same room. The fish were then allowed to acclimate inside the swim tunnel overnight at a low current speed of 15 cm s^−1^. The following morning the U_crit_ trial started and consisted of a stepwise increase in current speed of 15 cm s^−1^ every 30 minutes. At high speeds the fish would eventually struggle to maintain swimming positions and fall back on the rear grid. Fatigue was then defined as when a fish was unable to resume swimming even after tactile encouragement by the experimenter’s hand. At this point, time was noted, and the fish was removed and euthanized with a blow to the head. Immediately after, a blood sample was drawn via caudal puncture with a heparinized syringe and stored on ice momentarily until further processing. The weight and fork length was then recorded. The U_crit_ trial was continued until all eight fish had reached fatigue.

To measure ṀO_2_ in response to increasing swimming speeds in the U_crit_ trials, the water inflow was closed off for 20 minutes during each speed interval and flushed for the remaining 10 minutes, which ensured oxygen saturations above 85% throughout the trial. Closed measurement periods began 5 minutes into a new speed increment to provide some time for the fish to adjust swimming efforts and respiratory requirements, and thus ended 5 minutes prior to the onset of the next speed increment. Once the first fish had been removed due to fatigue, ṀO_2_ was no longer monitored on the remaining fish. All ṀO_2_ measurements thereby represented an average value of eight fish at defined activity levels (Hvas and Oppedal, 2019). Background respiration was not corrected as it was found to be undetectable at different temperatures in this large setup (Hvas *et al.,* 2017). Additionally, the tail beat frequency was measured in four random fish at each current speed by using camera observations to count the time required to perform 50 tail beats. Three replicate U_crit_ trials were performed at each temperature on both fed and fasted fish meaning that a total of 24 fish were tested per U_crit_ treatment group.

The sustained swim trials were performed after the associated U_crit_ trials had been completed at a specific temperature and feeding status on novel fish. The U_crit_ trials needed to be conducted prior to sustained swimming trials in order to determine the sustained swimming test speed, which was defined as 80% of the mean U_crit_ from the same cohort treatment. Similar to the U_crit_ trials, eight fish were moved to the tunnel in the afternoon to acclimate overnight at 15 cm s^−1^. The following morning the sustained swim trial started by increasing current speeds every 5 minutes by 15 cm s^−1^ until 80% U_crit_ had been reached. This speed was then maintained for 4 hours. If a fish became fatigued, it was removed and time was noted, while fish that did not become fatigued after 4 hours were noted to have completed the test. The fish were then euthanized whereafter weight and fork length were recorded. During the sustained swim trials, ṀO_2_ was measured in 30-minute intervals using 20-minute closed periods followed by 10 minutes of flushing. Furthermore, the tail beat frequency was measured every 30 minutes on four random fish, as in the U_crit_ trials. Three replicate sustained swim trials were performed at each temperature on both fed and fasted fish.

After the U_crit_ trials, blood sampled from fatigued fish were centrifuged in Eppendorf tubes at 6000 g for 5 minutes. The plasma supernatant was then transferred to new Eppendorf tubes and stored at −80°C until later analyses. Once all swim trials had been completed, plasma osmolality was measured with freeze point determination in 20 μl subsamples using a Fiske 210 Micro-Sample Osmometer (Advanced Instruments). The concentration of plasma cortisol was measured in 20 μl subsamples using an ELISA assay kit (standard range: 20–800 ng ml^−1^; IBL International GmbH). Finally, the concentration of plasma lactate, Na^+^ and Cl^−^ were measured in 65 μl subsamples with an ABL90 FLEX blood gas analyzer (Radiometer).

### Data analyses

The U_crit_ was calculated, according to Brett (1964), as follows:

$$ {\mathrm{U}}_{\mathrm{crit}}={\mathrm{U}}_{\mathrm{f}}+\frac{{\mathrm{t}}_{\mathrm{f}}{\mathrm{U}}_{\mathrm{i}}}{{\mathrm{t}}_{\mathrm{i}}}, $$

where U_f_ is the highest completed swimming speed, U_i_ is the increment between speeds (15 cm s^−1^), t_f_ is time endured at the last speed before reaching fatigue and t_i_ is the time interval between speeds (30 minutes). Since the cross-sectional area of the swim section being large relative to the size of the fish, solid blocking effects were not corrected for in the reported U_crit_ values, as this effect would be minimal (Bell and Terhune, 1970; Plaut, 2001).

The ṀO_2_ was calculated for each measurement period in the critical and sustained swim trials from the decrease in dissolved oxygen over time as follows:

\begin{align*} \dot{\mathrm{M}}{\mathrm{O}}_2=\frac{\frac{{\Delta \mathrm{O}}_2}{\Delta \mathrm{t}}\left({\mathrm{V}}_{\mathrm{sys}}-{\mathrm{V}}_{\mathrm{b}}\right)}{{\mathrm{M}}_{\mathrm{b}}}, \end{align*}

where ΔO_2_/Δt is the change in oxygen over time; V_sys_ is the volume of the swim tunnel system; V_b_ is the volume of the fish, assuming a density of 1 kg l^−1^; and M_b_ is the biomass of the fish. The CoT at specific swimming speeds could then be derived and was expressed as mg O_2_ kg^−1^ km^−1^. In addition, a quadratic regression was fitted to the U-shaped relationship between CoT and swimming speed in the U_crit_ trials, where the minimum of this function was defined as the U_opt_ for minimum CoT.

To assess the impact of fasting on body morphology, the condition factor of all fish was calculated as 100 (weight (g)/length(cm)^3^) (Ricker, 1975).

**Figure 1:**
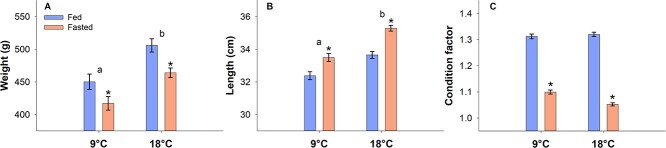
Size parameters. Statistical differences between temperatures regardless of feeding status are indicated with letters, and statistical differences between fed and fasted fish within a specific temperature are indicated with asterisks (two-way ANOVA with Hold–Sidak *post-hoc* analyses *P* < 0.05). *N* = 48 and data are mean ± s.e.m.

To test for statistical differences between feeding status and acclimation temperature on U_crit_, U_opt,_ size parameters and haematological parameters, a two-way ANOVA with the Hold–Sidak method for multiple comparison procedures was used after confirming normality and equal variance of the data with the Shapiro–Wilk test and the Brown–Forsythe test, respectively (SigmaPlot 14.5). To adhere to these test assumptions, it was necessary to perform a log transformation of the data for weight, osmolality, Na^+^ and Cl^−^. The ṀO_2_, CoT and tail beat frequency data were similarly analyzed with a two-way ANOVA to assess effects of treatment (feeding status plus temperature) at specific swimming speeds or time points in the U_crit_ and sustained swim trials, respectively. A *P*-value below 0.05 was considered significant in the statistical tests and data are reported as the mean ± s.e.m.

## Results

The 4-week fasting period did not result in any mortalities at either 9°C or 18°C, nor were any apparent changes in behaviours or incidences of injuries observed on the fish within the holding tanks.

Fish acclimated to 18°C were significantly heavier and longer than at 9°C regardless of feeding status (two-way ANOVA, DF = 191, *P* < 0.001 for both parameters). Condition factor was similar in fed fish across temperature (*P* = 0.491), but lower in fasted fish at 18°C compared with fasted fish at 9°C (*P* < 0.001). The fasting period reduced the weight significantly within both temperatures (9°C, *P* = 0.017; 18°C, *P* = 0.009) from 450 ± 12 to 417 ± 10 g at 9°C and from 506 ± 10 to 464 ± 7 g at 18°C, which corresponded to an average percentage weight loss of 7.3% and 8.3%, respectively (Fig. 1A). Meanwhile length increased significantly during fasting within temperature (*P* < 0.001 in both) from 32.4 ± 0.2 to 33.5 ± 0.2 cm at 9°C and from 33.7 ± 0.2 to 35.3 ± 0.2 cm at 18°C (Fig. 1B). These changes in weight and length resulted in significantly lower condition factors in fasted fish (*P* < 0.001 within both temperatures), from 1.31 ± 0.01 to 1.10 ± 0.01 at 9°C and from 1.32 ± 0.01 to 1.05 ± 0.01 at 18°C (Fig. 1C). The lowest measured condition factor on an individual fasted fish was 0.97 at 18°C.

**Figure 2:**
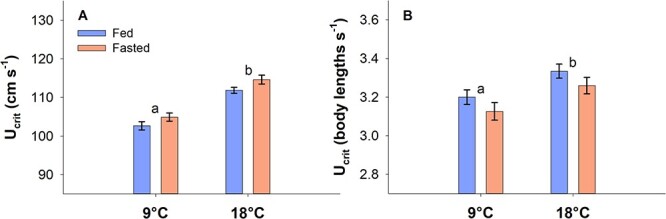
The critical swimming speed (U_crit_) expressed in cm s^−1^ (**A**) and body lengths s^−1^ (**B**). Statistical differences between temperatures regardless of feeding status are indicated with letters. Feeding status did not cause significant differences within either temperature (two-way ANOVA with Hold–Sidak *post-hoc* analyses, *P* < 0.05). *N* = 24 and data are mean ± s.e.m.

The U_crit_ was significantly higher at 18°C compared with at 9°C across feeding status both when expressed as cm s^−1^ and as body length s^−1^ (two-way ANOVA, DF = 95, *P* < 0.001) (Fig. 2A,B). However, the U_crit_ was unaffected by fasting period within either temperature when expressed in cm s^−1^ (9°C, *P* = 0.125; 18°C, *P* = 0.058) as well as in body length s^−1^ (9°C, *P* = 0.199; 18°C, *P* = 0.197) (Fig. 2A,B). Although not significantly different, fasted fish had a slightly higher U_crit_ when expressed in cm s^−1^ and a slightly lower U_crit_ when expressed in body length s^−1^, which can be ascribed to the increased fork lengths in fasted fish.

**Figure 3:**
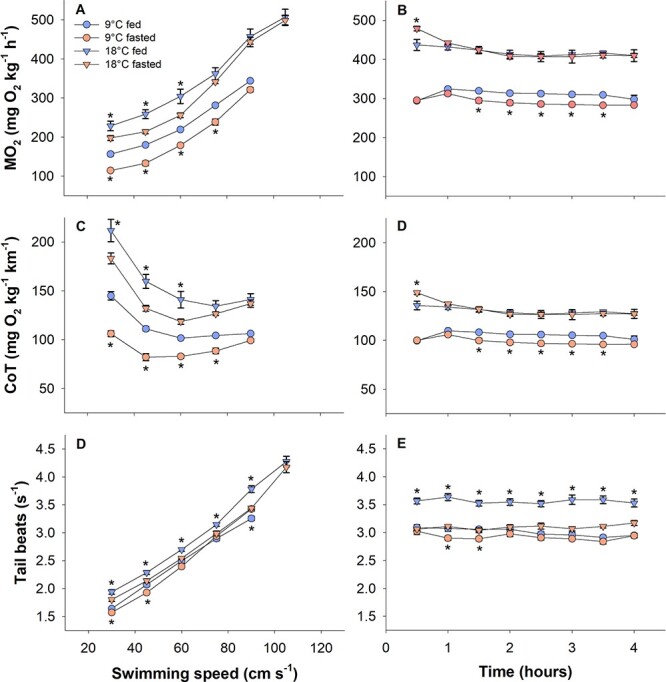
Metabolic rates (ṀO_2_), CoT and tailbeat frequencies in the U_crit_ (left panels) and sustained swim (right panels) trials. Statistical differences between fed and fasted fish within a specific temperature at different swim speeds or time points are indicated with asterisks (two-way ANOVA with Hold–Sidak *post-hoc* analyses, *P* < 0.05). *N* = 3 for ṀO_2_ and CoT measurements and *N* = 12 for tail beat frequencies. Data are mean ± s.e.m.

The sustained swim trials were based on the 80% U_crit_ of fish from the same temperature and feeding status. The specific sustained test speeds used were 82.1 cm s^−1^ (2.56 body lengths s^−1^) on fed fish at 9°C, 83.9 cm s^−1^ (2.50 body lengths s^−1^) on fasted fish at 9°C, 89.5 cm s^−1^ (2.67 body lengths s^−1^) on fed fish at 18°C and 91.7 cm s^−1^ (2.61 body lengths s^−1^) on fasted fish at 18°C. All fed and fasted fish tested at either temperature managed to complete 4 hours of swimming at 80% U_crit_ without becoming fatigued.

The ṀO_2_ increased with swimming speed and differed between all treatment groups in the U_crit_ trials (two-way ANOVA, DF = 59, *P* < 0.001), where fish at 18°C had a higher ṀO_2_ than at 9°C and fasted fish had a lower ṀO_2_ within both temperatures (Fig. 3A). Specific differences between fed and fasted fish in ṀO_2_ were found at 30, 45 and 60 cm s^−1^ at both temperatures while ṀO_2_ also differed at 75 cm s^−1^ within 9°C. At the highest test speeds, as the maximum aerobic metabolic rate was approached, the ṀO_2_ became similar between fed and fasted fish at both temperatures (Fig. 3A).

In the sustained swim trials, the ṀO_2_ differed between treatment groups (two-way ANOVA, DF = 95, *P* < 0.001). Fish at 18°C had a higher ṀO_2_ than fish at 9°C (*P* < 0.001), and within temperature fasted fish had a lower ṀO_2_ at 9°C (*P* < 0.001), but not at 18°C (*P* = 0.247) (Fig. 3B). Significant differences at specific time points between fed and fasted fish within temperature were found at 30 minutes for 18°C and at 90, 120, 150, 180 and 210 minutes for 9°C (Fig. 3B). The ṀO_2_ was generally constant over time within each treatment group during the 4-hour sustained swim test. One exception to this was in the fasted 18°C group where the 30-minute time point was higher than all other points, while the 60-minute time point differed from the 120-, 150- and 180-minute time points (Fig. 3B).

The CoT derived from the U_crit_ trials also differed between treatment groups and swimming speeds (two-way ANOVA, DF 59, *P* < 0.001) (Fig. 3C). Specifically, fish at 9°C had an overall lower CoT and fasted fish had a lower CoT within temperature. The CoT derived from the sustained swimming trials differed between groups in a similar manner to their ṀO_2_ response (two-way ANOVA, DF 59, *P* < 0.001) (Fig. 3D). Notably, CoT was lower at 9°C while fasted fish had lower CoT within 9°C (*P* < 0.001), but not within 18°C (*P* = 0.256) (Fig. 3D).

Tail beat frequency increased approximately linearly with swimming speed in the U_crit_ trials and remained constant over time during the 4-hour sustained swim trials within all treatment groups (Fig. 3E,F). Significant differences in tail beat frequency between treatments were found in the U_crit_ trials (two-way ANOVA, DF = 239, *P* < 0.001), where the most notable effect was that fed fish at 18°C had higher tail beat frequencies than fasted counterparts at 30, 45, 60, 75 and 90 cm s^−1^ (Fig. 3E). A similar tendency for higher tail beat frequencies in fed fish within 18°C was also observed in the sustained swim trials (two-way ANOVA, DF = 383, *P* < 0.001) (Fig. 3F).

**Figure 4:**
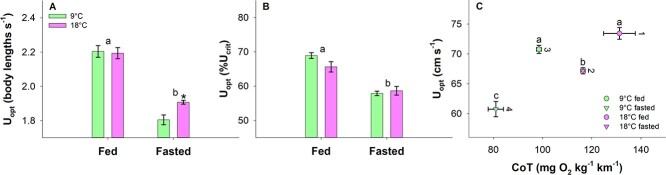
The optimal swimming speed (U_opt)_ for minimum CoT between groups expressed as body lengths s^−1^ (**A)**, a percentage of the critical swimming speed (U_crit)_ (**B**) and in cm s^−1^ versus the CoT (**C**). Statistical differences in (A) and (B) between fed and fasted fish across temperature are indicated with letters, and statistical differences between 9°C and 18°C within fed or fasted fish are indicated with asterisks. Vertical differences in U_opt_ in (C) are indicated with letters, while numbers indicate horizontal differences in CoT (two-way ANOVA with Hold–Sidak *post-hoc* analyses, *P* < 0.05). *N* = 3 and data are mean ± s.e.m.

The U_opt_ expressed in body lengths s^−1^, as a percentage of the U_crit_, and as cm s^−1^ versus CoT are shown in Fig. 4. Fasted fish had a significantly lower U_opt_ (body length s^−1^) than fed fish within both temperatures (two-way ANOVA, DF = 11, *P* < 0.001). Fed fish showed similar U_opt_ across temperatures (*P* = 0.802) whereas fasted fish were different and had a lower U_opt_ (body lengths s^−1^) at 9°C (*P* = 0.032) (Fig. 4A). Similarly, fasted fish had a lower U_opt_ (%U_crit_) within temperature (two-way ANOVA, DF = 11; 9°C, *P* < 0.001; 18°C, *P* = 0.002); however, both fed and fasted fish showed similar U_opt_ (%U_crit_) across temperatures (Fed, *P* = 0.068; Fasted, *P* = 0.0646) (Fig. 4B). The U_opt_ (cm s^−1^) showed the same patterns in statistical differences as the U_opt_ (body lengths s^−1^) (Fig. 4C). The minimum CoT differed in all comparisons between the four treatment groups with the fasted 9°C group being lowest and the 18°C fed group being highest (two-way ANOVA, DF = 11, *P* < 0.05) (Fig. 4C).

**Figure 5:**
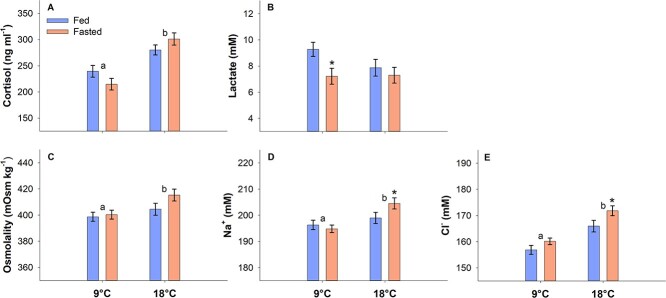
Haematological parameters. Blood samplings were done on exhausted fish immediately after U_crit_ measurements. Statistical differences between temperatures across feeding status are indicated with letters, and statistical differences between fed and fasted fish within a specific temperature are indicated with asterisks (two-way ANOVA with Hold–Sidak *post-hoc* analyses, *P* < 0.05). *N* = 24 and data are mean ± s.e.m.

The haematological parameters measured in fatigued fish after the U_crit_ trials are shown in Fig. 5. Plasma cortisol was higher at 18°C (two-way ANOVA, DF = 95, *P* < 0.001) and was unaffected by feeding status within each temperature (*P* = 0.863) (Fig. 5A). Significant differences in plasma lactate were only observed within 9°C where fed fish had higher lactate concentrations (*P* = 0.017) (Fig. 5B).

Plasma osmolality was significantly higher at 18°C (P = 0.013), while it was unaffected by feeding status within temperature (*P* = 0.122) (Fig. 5C). Both plasma Na^+^ and Cl^−^ concentrations were significantly higher at 18°C (*P* = 0.002 and *P* > 0.001, respectively), and fasted fish within 18°C had higher plasma Na^+^ and Cl^−^ concentrations (*P* = 0.041 and *P* = 0.023) (Fig. 5D,E).

## Discussion

### Weight loss and body condition

It was hypothesized that extended fasting at 18°C would assert a greater negative impact on body condition in Atlantic salmon owing to accelerated metabolic rates draining resources more quickly compared with at 9°C. Indeed, 4 weeks of fasting did cause a higher average weight loss and a greater reduction in condition factor at 18°C, coinciding with a higher ṀO_2_ at similar activity levels.

However, the difference in weight loss was surprisingly minor between the two temperatures being 7.3% and 8.3% at 9°C and 18°C, respectively. Meanwhile, using ṀO_2_ obtained at the lowest test speed in the U_crit_ swim trials as an indicator of routine metabolic costs irrespective of feeding status, fish at 18°C had a 57% higher ṀO_2_ than fish at 9°C. Factorial changes in functional rates in 10°C intervals are often expressed with the temperature quotient (Q_10_), and the increase in ṀO_2_ here corresponded to a Q_10_ of 1.7. Depending on acclimation history and section of the thermal niche assessed, resting metabolic rates in fish species generally increases with a Q_10_ of 1–3 (Lefevre, 2016), while the resting metabolic rates of thermally acclimated Atlantic salmon previously was found to increase with a Q_10_ of 2.1 between 8°C and 18°C (Hvas *et al.,* 2017). As such, if resting and routine energetic costs approximately doubles with a 10°C temperature increase, then weight loss over an extended fasting period should theoretically also double. However, similar proportional effects were clearly not found in the present study (i.e. 57% higher costs, but only a 1% difference in weight loss). This discrepancy may be explained by the ṀO_2_ only being an indirect measurement of aerobic metabolic rates (Nelson, 2016), and that the relationship between oxygen uptake and energy production can vary between environmental conditions (Salin *et al.,* 2015). Specifically, mitochondrial functioning becomes less efficient at higher temperatures due to impaired phosphorylation caused by increased proton leakage and decreased activity of complex I (Michaelsen *et al.,* 2021; Roussel and Voituron, 2020). For instance, Atlantic salmon had a lower ATP produced to oxygen consumed ratio in cardiac mitochondria when acclimated to 20°C compared with 12°C acclimation (Gerber *et al.,* 2021). Consequently, if less ATP is produced per oxygen at elevated temperatures, a higher ṀO_2_ is required to support similar metabolic demands as in lower temperatures. In this way temperature can cause a non-proportional relationship between ṀO_2_ and energy production in fish and other ectotherms (Salin *et al.,* 2015), and thus help explain a lower weight loss than otherwise expected from the proportionally greater increase in ṀO_2_ at 18°C. Assuming that ṀO_2_ and energy production were proportional across temperatures, a weight loss of approximately 12% instead of 8.3% would then have been expected at 18°C in the present study.

The reported weight loss of food-deprived Atlantic salmon in previous studies have generally been lower than the present one, likely owing to having used larger size classes, as mass specific energetic requirements decreases with size (Killen *et al.,* 2006; Oldham *et al.,* 2019), combined with lower temperatures. For instance, 8–9 weeks of fasting reduced weights by 6–7% (Hvas *et al.,* 2022; Reimers *et al.,* 1993) and 11–12 weeks of fasting reduced weights by 10–11% (Einen *et al.,* 1998; Lie and Huse, 1992), where these studies used fish ranging from 1.2 to 5 kg.

While losing weight the length increased over the 4-week fasting period. Similar continued length growth has previously been shown in food-deprived Atlantic salmon and indicates that vertebrae and muscle development may work independently of each other (Einen *et al.,* 1998; Hvas *et al.,* 2021b, 2022). Furthermore, continued length increases could prepare fish for accelerated muscle growth when feed becomes available again and thereby help facilitate compensatory growth (Ali *et al.,* 2003).

Exacerbated by continued length growth, the condition factor dropped substantially during fasting from 1.32 across temperature down to 1.10 and 1.05 at 9°C and 18°C, respectively. However, a state of severe starvation and malnourishment in Atlantic salmon is generally first associated with condition factors below 0.9 (Noble *et al.,* 2018). The fish in the present study could therefore have endured a longer fasting period before entering a stage of protein catabolization, which signifies severe starvation (Wang *et al.,* 2006). As such, considering the modest changes in weights and the resulting intermediate condition factors, 4 weeks of fasting was well within the tolerance limit of Atlantic salmon, even at a suboptimal elevated temperature of 18°C.

### Swimming capacity

The U_crit_ is highest in the upper ranges of the thermal niches in Atlantic salmon and other fish species studied, coinciding with higher maximum metabolic rates (Brett, 1964; Claireaux *et al.,* 2006; Hvas *et al.,* 2017; Yuen *et al.,* 2019). A higher U_crit_ at 18°C compared with at 9°C was therefore to be expected in the present study.

Interestingly, the U_crit_ was unaffected by the 4-week fasting period within both acclimation temperatures. In a separate study Atlantic salmon also maintained their full U_crit_ over a 4-week fasting period at 12°C (Hvas *et al.,* 2021b), and a similar result was therefore expected at 9°C owing to lower temperatures having a lesser impact on metabolic demands. However, an unaffected U_crit_ at 18°C was surprising as this elevated temperature represents suboptimal conditions for growth and behavioural preferences in this species (e.g. Johansson *et al.,* 2009; Kullgren *et al.,* 2013). In a study on food-deprived cod the U_crit_ was reduced in the food-deprived treatment, but their condition factor had decreased from 1.0 to 0.5 (Martínez *et al.,* 2004). While different species have different morphometrics, a halving of condition factor is a dramatic change compared with only a 20% reduction at 18°C in the present study. Hence, a more modest impact on body condition likely explains why U_crit_ remained unaffected in Atlantic salmon.

All fish tested managed to complete 4 hours of sustained swimming at 80% U_crit_ without becoming fatigued. The limit of sustained swimming in fed Atlantic salmon have consistently been found to be 80–85% U_crit_ at midrange temperatures of 12–13°C where speeds above 85% U_crit_ necessitates recruitment of anaerobic white muscle fibres and eventually causes fatigue (Beddow and McKinley, 1999; Hvas *et al.,* 2021a; Hvas and Oppedal, 2017). However, whether lower or higher temperatures would alter this threshold for aerobic swimming was previously unknown. It has been shown in other fish species that white muscles are recruited at lower swimming speeds in lower temperatures, which causes a reduction in sustained swimming capacity (Rome *et al.,* 1990; Sisson and Sidell, 1987; Taylor *et al.,* 1996). Similarly, in Atlantic salmon swimming at 3°C and 8°C, the transition to partially anaerobic burst and glide swimming occurs at lower speeds than at higher temperatures (Hvas *et al.,* 2017). However, in the present study, aerobic swimming limits were not yet impaired at 9°C.

The aetiology of fatigue can either be a failure to supply sufficient metabolites at the required rates (e.g. U_crit_ tests) or the depletion of metabolite supplies (e.g. fixed long-term velocity tests) (Jones, 1982). The latter being analogous to collapsing marathon runners ‘hitting the wall’ despite working within their normal aerobic limit. It was therefore hypothesized that fasted fish at 18°C eventually would fatigue in the sustained swim trials as food deprivation results in a greater exhaustion of resources at this temperature. However, as discussed above, the fasting regime at 18°C only caused a moderate reduction in body condition meaning that fish still were able to fuel long-term high intensity aerobic swimming. The 80% U_crit_ threshold for sustained swimming in Atlantic salmon can therefore now also be applied to a wider thermal interval and to fish that have experienced extended fasting periods.

The ṀO_2_ remained stable within each treatment group during 4 hours of sustained swimming, providing evidence for that swimming efforts indeed remained aerobic. If this was not the case, anaerobic metabolism would cause lactate to accumulate in muscles and blood since lactate clearance is slow in salmonids (Hvas *et al.,* 2021b; Schulte *et al.,* 1992). Furthermore, only 3 ATP are produced when glycogen is converted to lactate while 5 ATP are required to convert lactate back to glycogen (Burgetz *et al.,* 1998). Hence, if a significant anaerobic component was present, the increasing need to metabolize lactate at a cost deficit while swimming would drive up the ṀO_2_ until maximum rates were reached whereafter fatigue would be imminent.

Exhaustive exercise stress in salmonids causes substantial disturbances in osmotic, endocrine, and metabolic balances (Kieffer, 2000; Wood, 1991). The haematological parameters measured in the present study after the U_crit_ tests were therefore characteristic of fatigued Atlantic salmon in seawater (Hvas *et al*., 2018, 2020). However, since the U_crit_ was unaffected by food deprivation, only modest variations were found between treatment groups. Fish at 18°C were swimming for longer and at higher final speeds while achieving a substantially higher ṀO_2_, which facilitated a greater increase in plasma osmolality, Cl^−^ and Na^+^ compared with at 9°C owing to the osmorespiratory tradeoff of the fish gill in hyperosmotic environments (Hvas *et al.,* 2018; Sardella and Brauner, 2007). Interestingly, fasted fish at 18°C showed a greater ionic disturbance, which may be a subtle indicator that athletic performance was starting to become affected, as this would have some implications for repeat-swimming capabilities since recovery of ion homeostasis is particularly slow in seawater adapted Atlantic salmon (Hvas *et al.,* 2018, 2020). The higher cortisol values at 18°C were likely caused by a combination of increased swimming efforts and temperature, as the cortisol response to a fixed stressor also was elevated at 17°C compared with 9°C in a previous study on Atlantic salmon (Madaro *et al.,* 2018). Plasma lactate was unaffected by temperature, which suggests that similar anaerobic swimming efforts were made prior to fatigue meaning that the improved U_crit_ at 18°C was aerobically driven. However, fasted fish within 9°C had lower lactate levels, which could hint at lower functionality of white muscle fibres as shown for other food-deprived fish species (Beardall and Johnston, 1983; Martínez *et al.,* 2003). Although, considering that resting lactate levels should be <1 mM (e.g. Wood, 1991), and all treatment groups had greatly elevated values of >7 mM, this indicates preserved high capacities for anaerobic work following 4 weeks of fasting in Atlantic salmon.

### **Swimming energetics and U**_**opt**_

While the capacities for both aerobic and anaerobic swimming were preserved following extended fasting, some temperature-dependent changes in swimming energetics were observed. At low and intermediate swimming speeds from 30 to 60 cm s^−1^, fasted fish within both temperatures had a lower ṀO_2_. At 9°C this corresponded to an average reduction of 43.1 mg O_2_ kg^−1^ h^−1^ or 23.8% across speeds, and at 18°C a comparable absolute reduction of 41.4 mg O_2_ kg^−1^ h^−1^ was found, but a lower percentage reduction of 15.6% caused by higher basal costs. Similarly, the resting metabolic rate of Atlantic salmon after 3–4 weeks of fasting at 12°C was reduced by 22% (Hvas *et al.,* 2020), while other fish species also downregulate metabolic rates in response to food deprivation (Fu *et al.,* 2005; Mehner and Wieser, 1994).

These reductions in ṀO_2_ in fasted fish were unlikely to be an artefact of elevated ṀO_2_ in the fed control fish caused by specific dynamic action effects, as these fish had been fasting overnight for a minimum of 20 hours prior to measurements, which should have allowed for completion of most digestive processes (Handeland *et al.,* 2008; Storebakken *et al.,* 1999). Hence, lower ṀO_2_ in food-deprived fish should represent real adaptive responses to minimize energetic requirements that may involve a range of biochemical adjustments in gene expressions, decreased enzyme activities, reduced protein syntheses and improved efficiency of mitochondrial functioning in appropriate organs (Bermejo-Nogales *et al.,* 2015; Cassidy *et al.,* 2016; Méndez and Wieser, 1993; Salin *et al.,* 2018).

Interestingly, the ability to save energy during food deprivation was less pronounced at higher activity levels where the ṀO_2_ between fed and fasted fish converged, and this pattern occurred earlier at 18°C. As such, the ability to preserve energy appeared to be less efficient when fasting at higher temperatures in Atlantic salmon. In addition, the similar ṀO_2_ between fed and fasted fish at high activity levels suggests that maximum aerobic metabolic rats were unaffected, in agreement with an unaffected U_crit_. This observation is advantageous for food-deprived Atlantic salmon, as they can use less resources during resting and routine conditions while still being able to perform at their highest athletic ability, if needed.

The reduced ṀO_2_ in fasted fish resulted in lower CoT and U_opt_ at both temperatures, and these effects were greater at 9°C. This means that fasted Atlantic salmon in colder waters, in theory, will swim slower and at lower costs when foraging or migrating. For instance, based on the U_opt_ of the present study, a hypothetical 1000 km migration would increase from 16.3 to 19.1 days between fed and fasted fish at 9°C, and similarly increase from 15.7 to 17.2 days at 18°C. While using longer time to migrate a given distance, the overall energetic requirement will still be reduced in food-deprived Atlantic salmon, with 17.8% and 11.3% lower total costs at 9°C and 18°C, respectively. Moreover, regardless of feeding status, cruising at U_opt_ was 27.5% cheaper at 9°C compared with at 18°C. The maximum distance that can be covered by food restricted migratory Atlantic salmon will consequently be lower in warming marine environments.

## Conclusion

The Atlantic salmon is an environmentally flexible species that is able to handle and adapt to many of the diverse challenges it may encounter during its life cycle. This study exemplifies this flexibility as extended fasting was successfully endured with moderate effects on weight and body condition because of the ability to beneficially downregulate metabolic requirements, even at an elevated suboptimal temperature. Furthermore, despite of 4 weeks of food deprivation, Atlantic salmon maintained their full capacity for both aerobic and anaerobic swimming across temperatures as shown by a preserved U_crit_ and completion of the sustained swim trials.

However, some ecological implications may be considered with regards to swimming energetics in a warming climate in conjunction with other stressors. Elevated temperatures reduced the fasting-induced energetic savings, while inherently also driving up basal maintenance costs and thus draining resources more quickly. This could restrain migratory limits and have implications for survival, both during initial seaward travel in newly smoltified fish and upriver return in spawning adults. Specifically, seaward-bound juvenile Atlantic salmon are under substantial predation pressure owing to their small sizes and are also at high risk of parasite infections dispersed from aquaculture farms when travelling through the fjords and coastal zones (Johnsen *et al.,* 2020; Thorstad *et al.,* 2012). Rapid growth at sea is therefore crucial to ensure survival, but this task could become more difficult in warmer marine environments, particularly if fish also have to cope with the energetic burden of parasites (Hvas and Bui, 2022; Susdorf *et al.,* 2018). Furthermore, Atlantic salmon become anorexic when migrating back to their spawning grounds (Kadri *et al.,* 1995). Provided lower initial body conditions (e.g. Susdorf *et al.,* 2018) together with increased travel costs at higher temperatures (present study), while diverting substantial resources on gonad development (Hendry and Beall, 2004), this could lead to shorter and less prolific spawning events as well as reducing the number of surviving repeat-spawning adult Atlantic salmon.

## Acknowledgements

The author thanks the technical staff at the Matre Research station for excellent animal husbandry, Karen Anita Kvestad for performing haematological analyses and Tone Vågseth for assisting with the swim tunnel setup.

## Funding

This work was funded by the Research Council of Norway through the projects SFI Exposed (237790) and Fastwell (295200).

## Supplementary material

Supplementary material is available at *Conservation Physiology* online.

## Contributions

M.H. conceived and performed the experiment, analyzed the data and wrote the manuscript.

## Data availability

Raw data is available in a supplementary file.

## Conflict of Interest

The authors declare that they have no competing interests.

## Supplementary Material

Web_Material_coac037

## References

[ref1] Ali M , Nicieza A, Wootton RJ (2003) Compensatory growth in fishes: a response to growth depression. Fish Fish 4: 147–190.

[ref2] Beamish FWH (1978) Swimming capacity. In WS Hoar, DJ Randall, eds, Fish Physiology. Academic Press, New York, NY, pp. 101–187

[ref3] Beardall CH , Johnston IA (1983) Muscle atrophy during starvation in a marine teleost. Eur J Cell Biol 29: 209–217. 6832167

[ref4] Beddow TA , McKinley RS (1999) Importance of electrode positioning in biotelemetry studies estimating muscle activity in fish. J Fish Biol 54: 819–831.

[ref5] Bell WH , Terhune LDB (1970) Water tunnel design for fisheries research. Fisheries Research Board of Canada, Technical Report No. 195.

[ref6] Bermejo-Nogales A , Calduch-Giner JA, Pérez-Sánchez J (2015) Unraveling the molecular signatures of oxidative phosphorylation to cope with the nutritionally changing metabolic capabilities of liver and muscle tissues in farmed fish. PLoS One 10: e0122889. 25875231 10.1371/journal.pone.0122889 PMC4398389

[ref7] Brett JR (1964) The respiratory metabolism and swimming performance of young sockeye salmon. J Fish Res Board Can 21: 1183–1226.

[ref8] Brett JR (1971) Energetic responses of salmon to temperature. A study of some thermal relations in the physiology and freshwater ecology of sockeye salmon (*Oncorhynchus nerka*). Am Zool 11: 99–113.

[ref9] Burgetz IJ , Rojas-Vargas A, Hinch SG, Randall DJ (1998) Initial recruitment of anaerobic metabolism during submaximal swimming in rainbow trout (*Oncorhynchus mykiss*). J Exp Biol 201: 2711–2721. 9732326 10.1242/jeb.201.19.2711

[ref10] Cassidy AK , Saulnier RJ, Lamarre SG (2016) Adjustments of protein metabolism in fasting Arctic Charr, Salvelinus alpinus. PLoS One 11: e0153364. 27096948 10.1371/journal.pone.0153364 PMC4838323

[ref11] Claireaux G , Couturier C, Groison A-L (2006) Effect of temperature on maximum swimming speed and cost of transport in juvenile European sea bass (*Dicentrarchus labrax*). J Exp Biol 209: 3420–3428. 16916977 10.1242/jeb.02346

[ref12] Clark TD , Sandblom E, Jutfelt F (2013) Aerobic scope measurements of fishes in an era of climate change: respirometry, relevance and recommendations. J Exp Biol 216: 2771–2782. 23842625 10.1242/jeb.084251

[ref13] Cotterell SP , Wardle CS (2004) Endurance swimming of diploid and triploid Atlantic salmon. J Fish Biol 65: 55–68.

[ref14] Dadswell M , Spares A, Reader J, McLean M, McDermott T, Samways K, Lilly J (2021) The decline and impending collapse of the Atlantic salmon (*Salmo salar*) population in the North Atlantic Ocean: a review of possible causes. Rev Fish Sci Aquac 30: 215–258.

[ref15] Drenner SM , Clark TD, Whitney CK, Martins EG, Cooke SJ, Hinch SG (2012) A synthesis of tagging studies examining the behaviour and survival of anadromous salmonids in marine environments. PLoS One 7: e31311. 22431962 10.1371/journal.pone.0031311 PMC3303779

[ref16] Einen O , Waagan B, Thomassen MS (1998) Starvation prior to slaughter in Atlantic salmon (*Salmo salar*). Aquaculture 166: 85–104.

[ref17] Eliason EJ , Farrell AP (2016) Oxygen uptake in Pacific salmon *Oncorhynchus* spp.: when ecology and physiology meet. J Fish Biol 88: 359–388. 26577675 10.1111/jfb.12790

[ref18] Farrell AP (2007) Cardiorespiratory performance during prolonged swimming tests with salmonids: a perspective on temperature effects and potential analytical pitfalls. Philos Trans R Soc Lond B Biol Sci 362: 2017–2030. 17553773 10.1098/rstb.2007.2111 PMC2442863

[ref19] Fu SJ , Xie XJ, Cao ZD (2005) Effect of fasting on resting metabolic rate and postprandial metabolic response in Silurus meridionalis. J Fish Biol 67: 279–285.

[ref20] Gamperl AK , Ajiboye OO, Zanuzzo FS, Sandrelli RM, Peroni EFC, Beemelmanns A (2020) The impacts of increasing temperature and moderate hypoxia on the production characteristics, cardiac morphology and haematology of Atlantic salmon (*Salmo salar*). Aquaculture 519: 734874.

[ref21] Gerber L , Clow KA, Gamperl AK (2021) Acclimation to warm temperatures has important implications for mitochondrial function in Atlantic salmon (*Salmo salar*). J Exp Biol 224: jeb236257. 33288533 10.1242/jeb.236257

[ref22] Green J , Farwell M (1971) Winter habits of the cunner,Tautogolabrus adspersus(Walbaum 1792), in Newfoundland. Can J Res Sect D Zool Sci 49: 1497–1499.

[ref23] Handeland SO , Björnsson BT, Arnesen AM, Stefansson SO (2003) Seawater adaptation and growth of post-smolt Atlantic salmon (*Salmo salar*) of wild and farmed strains. Aquaculture 220: 367–384.

[ref24] Handeland SO , Imsland AK, Stefansson SO (2008) The effect of temperature and fish size on growth, feed intake, food conversion efficiency and stomach evacuation rate of Atlantic salmon post-smolts. Aquaculture 283: 36–42.

[ref25] Hendry AP , Beall E (2004) Energy use in spawning Atlantic salmon. Ecol Freshw Fish 13: 185–196.

[ref26] Hevrøy EM , Tipsmark CK, Remø SC, Hansen T, Fukuda M, Torgersen T, Vikeså V, Olsvik PA, Waagbø R, Shimizu M (2015) Role of the GHIGF-1 system in Atlantic salmon and rainbow trout postsmolts at elevated water temperature. Comp Biochem Physiol A Mol Integr Physiol 188: 127–138. 26144599 10.1016/j.cbpa.2015.06.030

[ref27] Hvas M , Bui S (2022) Energetic costs of ectoparasite infection in Atlantic salmon. J Exp Biol 225: jeb243300. 34931653 10.1242/jeb.243300

[ref28] Hvas M , Folkedal O, Imsland A, Oppedal F (2017) The effect of thermal acclimation on aerobic scope and critical swimming speed in Atlantic salmon *Salmo salar*. J Exp Biol 220: 2757–2764. 28507190 10.1242/jeb.154021

[ref29] Hvas M , Folkedal O, Oppedal F (2021a) What is the limit of sustained swimming in Atlantic salmon post smolts? Aquacult Environ Interact 13: 189–198.

[ref30] Hvas M , Nilsen TO, Oppedal F (2018) Oxygen uptake and osmotic balance of Atlantic salmon in relation to exercise and salinity acclimation. Front Mar Sci 5: 368.

[ref31] Hvas M , Nilsson J, Vågseth T, Nola V, Fjelldal PG, Hansen TJ, Oppedal F, Stien LH, Folkedal O (2022) Full compensatory growth before harvest and no impact on fish welfare in Atlantic salmon after an 8-week fasting period. Aquaculture 546: 737415.

[ref32] Hvas M , Oppedal F (2017) Sustained swimming capacity of Atlantic salmon. Aquacult Environ Interact 9: 361–369.

[ref33] Hvas M , Oppedal F (2019) Influence of experimental set-up and methodology for measurements of metabolic rates and critical swimming speed in Atlantic salmon *Salmo salar*. J Fish Biol 95: 893–902. 31265133 10.1111/jfb.14087

[ref34] Hvas M , Stien LH, Oppedal F (2020) The metabolic rate response to feed withdrawal in Atlantic salmon post-smolts. Aquaculture 529: 735690.

[ref35] Hvas M , Stien LH, Oppedal F (2021b) The effect of fasting period on swimming performance, blood parameters and stress recovery in Atlantic salmon post smolts. Comp Biochem Physiol A Physiol 255: 110913. 10.1016/j.cbpa.2021.110913 33524618

[ref36] Jensen AJ , Karlsson PF, Hansen LP, Østborg GM, Hindar K (2014) Origin and life history of Atlantic salmon (*Salmo salar*) near their northernmost oceanic limit. Can J Fish Aquat Sci 71: 1740–1746.

[ref37] Johansson D , Ruohonen K, Juell J-E, Oppedal F (2009) Swimming depth and thermal history of individual Atlantic salmon (*Salmo salar* L.) in production cages under different ambient temperature conditions. Aquaculture 290: 296–303.

[ref38] Johnsen IA , Harvey A, Sævik PN, Sandvik AD, Ugedal O, Ådlandsvik B, Wennevik V, Glover KA, Karlsen Ø (2020) Salmon lice-induced mortality of Atlantic salmon during post-smolt migration in Norway. ICES J Mar Sci 78: 142–154.

[ref39] Johnston IA , Goldspink G (1973) Some effects of prolonged starvation on the metabolism of the red and white myotomal muscles of the plaice *Pleuronectes platessa*. Mar Biol 19: 348–353.

[ref40] Jones DR (1982) Anaerobic exercise in teleost fish. Can J Zool 60: 1131–1134.

[ref41] Kadri S , Metcalfe NB, Huntingford FA, Thorpe JE (1995) What controls the onset of anorexia in maturing adult female Atlantic salmon? Funct Ecol 9: 790–797.

[ref42] Kieffer JD (2000) Limits to exhaustive exercise in fish. Comp Biochem Physiol A 126: 161–179. 10.1016/s1095-6433(00)00202-6 10938136

[ref43] Killen SS , Costa I, Brown JA, Gamperl AK (2007) Little left in the tank: metabolic scaling in marine teleosts and its implications for aerobic scope. Proc Biol Sci 274: 431–438. 17164208 10.1098/rspb.2006.3741 PMC1702384

[ref44] Krkošek M , Ford JS, Morton A, Lele S, Myers RA, Lewis MA (2007) Declining wild salmon populations in relation to parasites from farm salmon. Science 318: 1772–1775. 18079401 10.1126/science.1148744

[ref45] Kullgren A , Jutfelt F, Fontanillas R, Sundell K, Samuelsson L, Wiklander K, Kling P, Koppe W, Larson DGJ, Björnsson BT et al. (2013) The impact of temperature on the metabolome and endocrine metabolic signals in Atlantic salmon (*Salmo salar*). Comp Biochem Physiol A 164: 44–53. 10.1016/j.cbpa.2012.10.005 23051589

[ref46] Lacroix GL (2013) Population-specific ranges of oceanic migration for adult Atlantic salmon (*Salmo salar*) documented using pop-up satellite archival tags. Can J Fish Aquat Sci 70: 1011–1030.

[ref47] Lefevre S (2016) Are global warming and ocean acidification conspiring against marine ectotherms? A meta-analysis of the respiratory effects of elevated temperature, high CO_2_ and their interaction. Conserv Physiol 4: cow009. 27382472 10.1093/conphys/cow009 PMC4922249

[ref48] Lie Ø , Huse J (1992) The effect of starvation on the composition of Atlantic salmon (*Salmo salar*). Fisk Dir Skr Ernæring 5: 11–16.

[ref49] Loughna PT , Goldspink G (1984) The effects of starvation upon protein turnover in red and white myotomal muscle of rainbow trout, Salmo gairdneri Richardson. J Fish Biol 25: 223–230.

[ref50] Madaro A , Folkedal O, Maiolo S, Alvanopoulou M, Olsen RE (2018) Effects of acclimation temperature on cortisol and oxygen consumption in Atlantic salmon (*Salmo salar*) post-smolt exposed to acute stress. Aquaculture 497: 331–335.

[ref51] Martínez M , Bédard M, Dutil J-D, Guderley H (2004) Does condition of Atlantic cod (*Gadus morhua*) have a greater impact upon swimming performance atUcrit or sprint speeds? J Exp Biol 207: 2979–2990. 15277553 10.1242/jeb.01142

[ref52] Martínez M , Guderley H, Dutil J-D, Winger PD, Walsh SJ (2003) Condition, prolonged swimming performance and muscle metabolic capacities of cod (*Gadus morhua*). J Exp Biol 206: 503–511. 12502771 10.1242/jeb.00098

[ref53] McCue MD (2010) Starvation physiology: reviewing the different strategies animals use to survive a common challenge. Comp Biochem Physiol A Physiol 156: 1–18. 10.1016/j.cbpa.2010.01.002 20060056

[ref54] Mehner T , Wieser W (1994) Energetics and metabolic correlates of starvation in juvenile perch (*Perca fluviatilis*). J Fish Biol 45: 325–333.

[ref55] Méndez G , Wieser W (1993) Metabolic responses to food deprivation and refeeding in juveniles of *Rutilus rutilus* (Teleostei: *Cyprinidae*). Environ Biol Fishes 36: 73–81.

[ref56] Michaelsen J , Fago A, Bundgaard A (2021) High temperature impairs mitochondrial function in rainbow trout cardiac mitochondria. J Exp Biol 224: jeb242382. 33758025 10.1242/jeb.242382

[ref57] Miller KM , Schulze AD, Ginther N, Li S, Patterson DA, Farrell AP, Hinch SG (2009) Salmon spawning migration: metabolic shifts and environmental triggers. Comp Biochem Physiol 4: 75–89. 10.1016/j.cbd.2008.11.002 20403740

[ref58] Morgan IJ , McDonald DG, Wood CM (2001) The cost of living for freshwater fish in a warmer, more polluted world. Glob Chang Biol 7: 345–355.

[ref59] Morgan IJ , Metcalfe NB (2001) Deferred costs of compensatory growth after autumnal food shortage in juvenile salmon. Proc R Soc B 268: 295–301. 10.1098/rspb.2000.1365 PMC1088606 11217901

[ref60] Nelson JA (2016) Oxygen consumption rate v. rate of energy utilization of fishes: a comparison and brief history of the two measurements. J Fish Biol 88: 10–25. 26768970 10.1111/jfb.12824

[ref61] Noble C , Gismervik K, Iversen MH, Kolarevic J, Nilsson J, Stien LH, Turnbull JF (2018) Welfare indicators for farmed Atlantic Salmon: tools for assessing fish welfare. 351.

[ref62] Oldham T , Nowak B, Hvas M, Oppedal F (2019) Metabolic and functional impacts of hypoxia vary with size in Atlantic salmon. Comp Biochem Physiol A Mol Integr Physiol 231: 30–38. 30690152 10.1016/j.cbpa.2019.01.012

[ref63] Pardo SA , Bolstad GH, Dempson JB, April J, Jones JA, Raab D, Hutchings JA (2021) Trends in marine survival of Atlantic salmon populations in eastern Canada. ICES J Mar Sci 78: 2460–2473.

[ref64] Parrish DL , Behnke RJ, Gephard SR, McCormick SD, Reeves GH (1998) Why aren’t there more Atlantic salmon (*Salmo salar*)? Can J Fish Aquat Sci 55: 281–287.

[ref65] Plaut I (2001) Critical swimming speed: its ecological relevance. Comp Biochem Physiol A Mol Integr Physiol 131: 41–50. 11733165 10.1016/s1095-6433(01)00462-7

[ref66] Porter WP , Gates DM (1969) Thermodynamic equilibria of animals with environment. Ecol Monogr 39: 227–244.

[ref67] Reddin DG (1985) Atlantic salmon (*Salmo salar*) on and east of the grand bank. J Northwest Atl Fish Sci 6: 157–164.

[ref68] Reimers E , Kjørrefjord AG, Stavøstrand SM (1993) Compensatory growth and reduced maturation in second sea winter farmed Atlantic salmon following starvation in February and march. J Fish Biol 43: 805–810.

[ref69] Remen M , Solstorm F, Bui S, Klebert P, Vågseth T, Solstorm D, Hvas M, Oppedal F (2016) Critical swimming speed in groups of Atlantic salmon *Salmo salar*. Aquacult Environ Interact 8: 659–664.

[ref70] Ricker WE (1975) Computation and interpretation of biological statistics of fish populations. Bull Fish Res Board Can 191: 1–382.

[ref71] Rome LC , Funke RP, Alexander RL (1990) The influence of temperature on muscle velocity and sustained performance in swimming carp. J Exp Biol 154: 163–178. 2277258 10.1242/jeb.154.1.163

[ref72] Roussel D , Voituron Y (2020) Mitochondrial costs of being hot: effects of acute thermal change on liver bioenergetics in toads (*Bufo bufo*). Front Physiol 11: 153. 32218742 10.3389/fphys.2020.00153 PMC7078649

[ref73] Salin K , Auer SK, Rey B, Selman C, Metcalfe NB (2015) Variation in the link between oxygen consumption and ATP production, and its relevance for animal performance. Proc R Soc B 282: 20151028. 10.1098/rspb.2015.1028 PMC4528520 26203001

[ref74] Salin K , Villasevil EM, Anderson GJ, Auer SK, Selman C, Hartley RC, Mullen W, Chinopoulos C, Metcalfe NB (2018) Decreased mitochondrial metabolic requirements in fasting animals carry an oxidative cost. Funct Ecol 32: 2149–2157. 30333678 10.1111/1365-2435.13125 PMC6175143

[ref75] Sardella BA , Brauner CJ (2007) The osmo-respiratory compromise in fish. In MN Fernandes, FT Rantin, ML Glass, BG Kapoor, eds, Fish Respiration and Environment. CRC Press, Boca Raton, FL, pp. 147–165

[ref76] Scarabello M , Wood CM, Heigenhauser GJF (1991) Glycogen depletion in juvenile rainbow trout as an experimental test of the oxygen debt hypothesis. Can J Zool 69: 2562–2568.

[ref77] Schulte PM , Moyes CD, Hochachka PW (1992) Integrating metabolic pathways in post-exercise recovery of white muscle. J Exp Biol 166: 181–195. 1602273 10.1242/jeb.166.1.181

[ref78] Sisson JEIII , Sidell BD (1987) Effect of thermal acclimation on muscle fiber recruitment of swimming striped bass (*Morone saxatilis*). Physiol Zool 60: 310–320.

[ref79] Steffensen JF , Johansen K, Bushnell PG (1984) An automated swimming respirometer. Comp Biochem Physiol A Physiol 79: 437–440.

[ref80] Storebakken T , Kvien IS, Shearer KD, Grisdale-Helland B, Helland SJ (1999) Estimation of gastrointestinal evacuation rate in Atlantic salmon (*Salmo salar*) using inert markers and collection of faeces by sieving: evacuation of diets with fish meal, soybean meal or bacterial meal. Aquaculture 172: 291–299.

[ref81] Susdorf R , Salama NKG, Todd CD, Hillman RJ, Elsmere P, Lusseau D (2018) Context-dependent reduction in somatic condition of wild Atlantic salmon infested with sea lice. Mar Ecol Prog Ser 606: 91–104.

[ref82] Taylor SE , Egginton S, Taylor EW (1996) Seasonal temperature acclimatisation of rainbow trout: cardiovascular and morphometric influences on maximal sustainable exercise level. J Exp Biol 199: 835–845. 9318615 10.1242/jeb.199.4.835

[ref83] Thorstad EB , Whoriskey F, Uglem I, Moore A, Rikardsen AH, Finstad B (2012) A critical life stage of the Atlantic salmon *Salmo salar*: behaviour and survival during the smolt and initial post-smolt migration. J Fish Biol 81: 500–542. 22803722 10.1111/j.1095-8649.2012.03370.x

[ref84] Valiente AG , Juanes F, Garcia-Vazquez EG (2011) Increasing regional temperatures associated with delays in Atlantic salmon sea-run timing at the southern edge of the European distribution. Trans Am Fish Soc 140: 367–373.

[ref85] Van Ginneken VJT , Antonissen E, Müller UK, Booms R, Eding E et al. (2005) Eel migration to the Sargasso: remarkably high swimming efficiency and low energy costs. J Exp Biol 208: 1329–1335. 15781893 10.1242/jeb.01524

[ref86] Wade NM , Clark TD, Maynard BT, Atherton S, Wilkinson RJ, Smullen RP, Taylor RS (2019) Effects of an unprecedented summer heatwave on the growth performance, flesh colour and plasma biochemistry of marine cage-farmed Atlantic salmon (*Salmo salar*). J Therm Biol 80: 64–74. 30784489 10.1016/j.jtherbio.2018.12.021

[ref87] Wang T , Hung CCU, Randall DJ (2006) The comparative physiology of food deprivation: from feast to famine. Annu Rev Physiol 68: 223–251. 16460272 10.1146/annurev.physiol.68.040104.105739

[ref88] Weihs D (1973) Optimal fish cruising speed. Nature 245: 48–50.

[ref89] Wilson RW , Egginton S (1994) Assessment of maximum sustainable swimming performance in rainbow trout (*Oncorhynchus mykiss*). J Exp Biol 192: 299–305. 9317845 10.1242/jeb.192.1.299

[ref90] Wood CM (1991) Acid-base and ion balance, metabolism, and their interactions, after exhaustive exercise in fish. J Exp Biol 160: 285–308.

[ref91] Yuen JW , Dempster T, Oppedal F, Hvas M (2019) Physiological performance of ballan wrasse (*Labrus bergylta*) at different temperatures and its implication for cleaner fish usage in salmon aquaculture. Biol Control 135: 117–123.

