# Supplementary material for: Swimming energetics of Atlantic salmon in relation to extended fasting at different temperatures
Source: Conserv Physiol. 2022 Jun 17;10(1):coac037. doi: 10.1093/conphys/coac037 (PMC9208137; doi:10.1093/conphys/coac037)

**1. Conceptual drawing of the swim tunnel:** ID is internal diameter. In addition to the details shown here, the setup can be fully closed off periodically for use as a respirometer. In the present study, an oxygen sensor was deployed next to the camera down stream of the rear grid. This diagram is borrowed from Remen et al. (2016): https://doi.org/10.3354/aei00207

**2. Photo of the swim tunnel:** Here the tunnel is being setup in our wet lab at the Matre research station. The tunnel can be assembled either with one of two of the white swim sections. In the present study only one swim section was used to provide better biomass to volume ratios for oxygen uptake measurements. Fish tested in the swim tunnel from the various treatment groups were being kept in the large holding tanks seen on the photo.


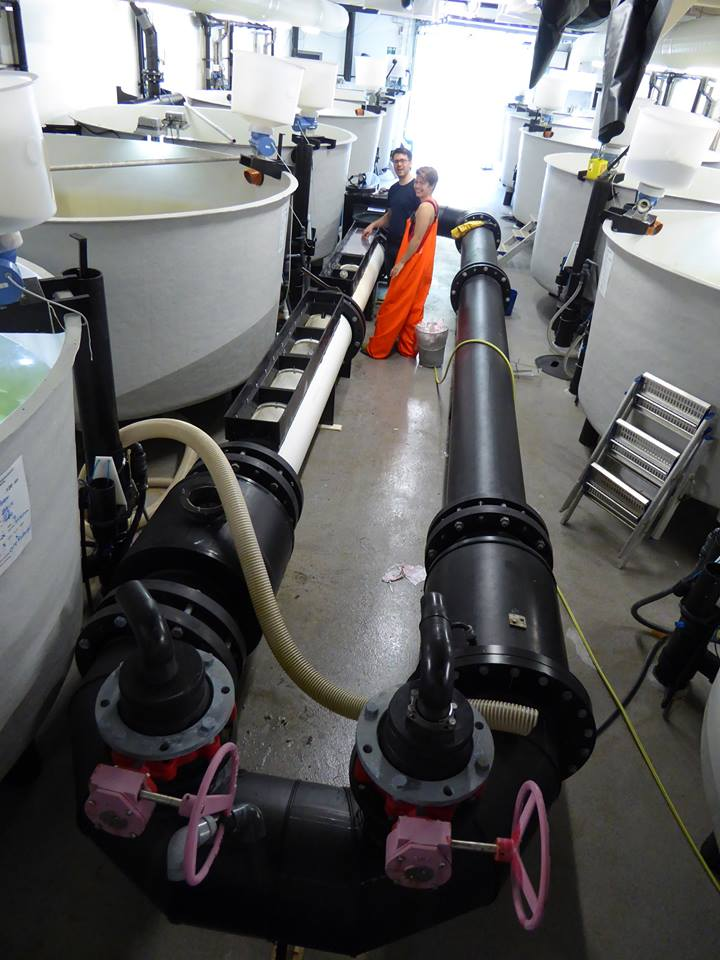


**3. Photo of the swim tunnel after final assemblage.** The person provides a sense of scale.


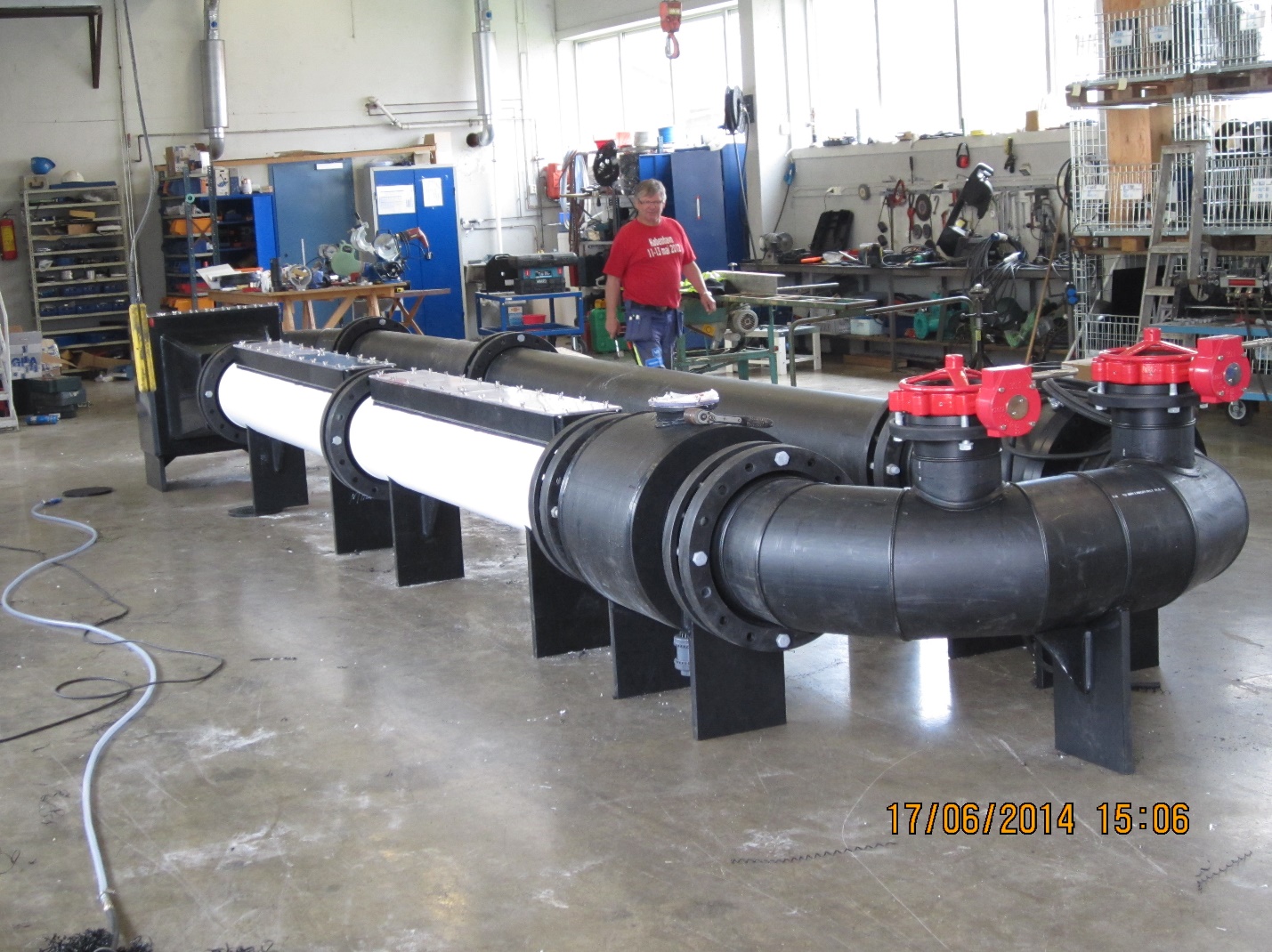


**4. Photo of the resting tank and reduction cone.** The removeable lid allows access to the flow-straightener with honeycomb shaped cells.

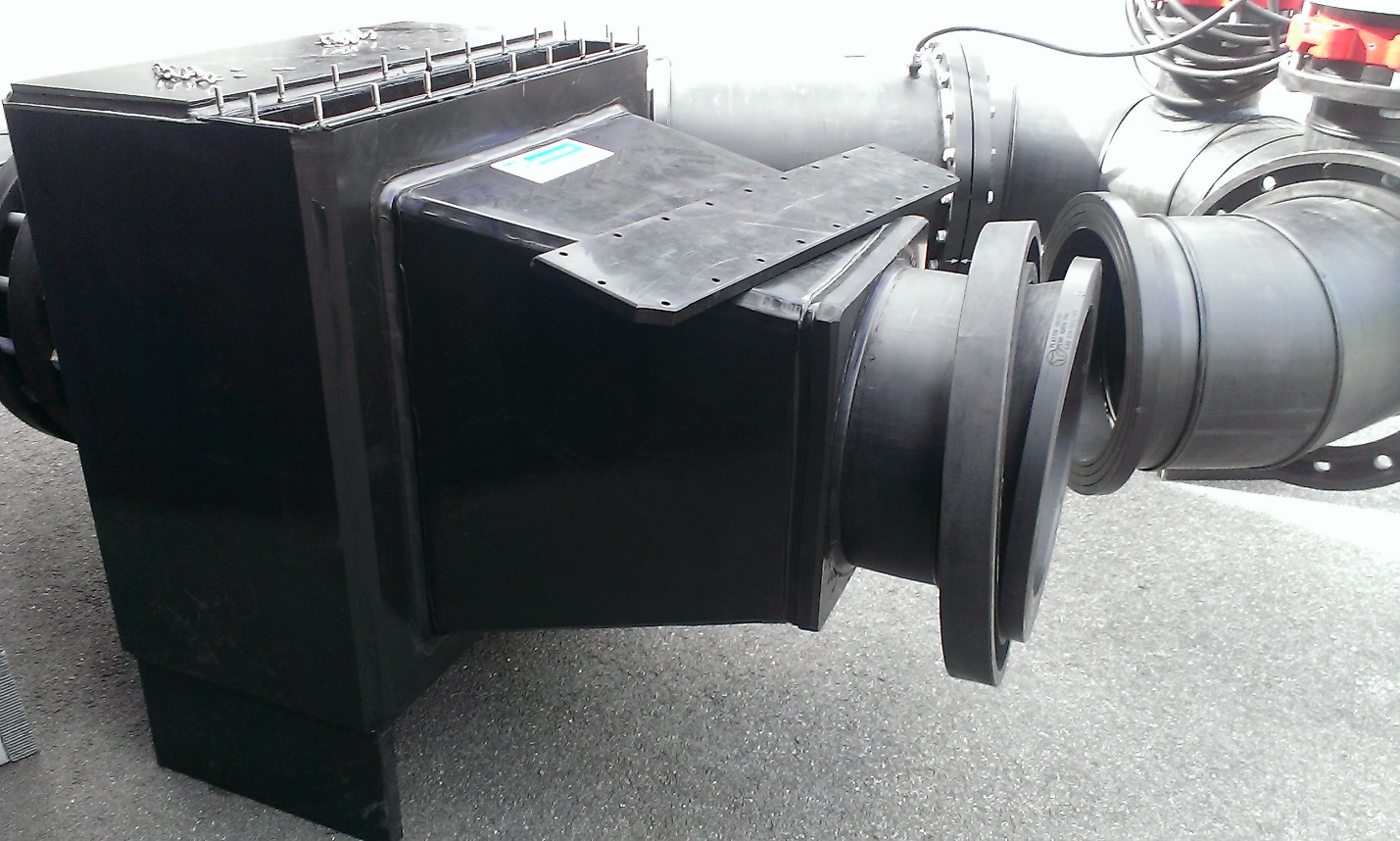

Supplement: Web_Material_coac037 [file web_material_coac037.zip › Supp. Swim tunnel setup.docx]
